# Physiological Reports 2020 Reviewers

**DOI:** 10.14814/phy2.14756

**Published:** 2021-03-05

The following experts completed reviews for *Physiological Reports* in 2020. We thank the reviewers for their important service to the community, especially during a challenging year.

Abassi, Zaid

Abbasi, Adeel

Abd El‐Mottaleb, Nashwa

Adams, William

Aeles, Jeroen

Affourtit, Charles

Ahmed, Mavra

Ahmed, Omyma G.

Akerman, Ashley

Alam, Uazman

Alexander, Matt

Alexander, R. Todd

Allen, Timothy

Alogna, Alessio

Alonso‐Vale, Maria Isabel

Alonso, Laura

Alpini, Gianfranco

Amann, Markus

Amasheh, Salah

Anderson, Greg

Ando, Ryosuke

Andrade, Francisco

Angus, Ja

Arslan, Mustafa

Artioli, Guilherme Giannini

Au, Jason

Austin, Eric Douglas

Azab, Mohammad

Badenhorst, Claire

Bagchi, Debasis

Bailey, Matthew

Baker, Sarah

Balslev, Daniela

Barry, Jonathan

Barstow, Thomas

Barthélemy, Dorothy

Bastarache, Julie

Bates, Carlton

Bates, Melissa

Batterham, Alan

Baum, Michel

Baumert, Philipp

Baumgarner, Bradley

Baynard, Tracy

Bebarova, Marketa

Beetham, Kassia

Behera, Jyotirmaya

Behm, David G.

Behnke, Bradley

Beiter, Thomas

Bergman, Bryan

Bertram, Hanne Christine

Beyer, Andreas

Bhalla, Vivek

Bharati, Jaya

Bianco, Antonino

Billinger, Sandra

Birch‐Machin, Mark

Bjorling, Dale

Bobe, Régis

Boccia, Gennaro

Boesen, Erika

Bollmann, Johann

Bonafiglia, Jacob

Borradaile, Nica

Bosc, Laura

Bourjeily, Ghada

Boyd‐Shiwarski, Cary

Bradbury, Neil

Brandebourg, Terry

Brassard, Patrice

Briant, Linford

Brocherie, Franck

Brook, Matthew

Brooks, Susan

Brown, Angus

Brownstein, Callum

Bruce, Kimberly

Bruscia, Emanuela

Bryant, Andrew

Buehler, Paul

Butcher, Joshua

Button, Duane

Calloe, Kirstine

Cameron, Ian

Camic, Clayton

Cammarota, Gianmaria

Cao, Yuhai

Capella, Marcia A. M.

Carattino, Marcelo

Cardozo, Christopher

Cardozo, Licy Yanes

Carter, Carol

Casuso, Rafael

Ceccatto, Vania M.

Cerda‐Kohler, Hugo

Chablani, Sumedha

Challet, Etienne

Chan, Stephen

Chang, Chen‐Kang

Charkoudian, Nisha

Chaseling, Georgia

Chen, Michael

Chen, Shiyou

Chen, Trevor

Cho, Yoshitake

Choi, Julia

Chopyk, Daniel

Choudhary, Gaurav

Chtourou, Hamdi

Clarke, Lane

Clifford, Bethan

Cobley, James

Coe, Imogen

Collins, James

Conde, Silvia

Connor, James

Copp, Steven

Cora, Michelle C.

Cordeiro, Jonathan

Cote, Anita T.

Couet, Jacques

Cowles, Robert

Crowley, Steven

Crowley, Steven D.

Csipo, Tamas

Cuellar, Carlos

Cummings, Bethany

Cummins, Theodore

D'Aout, Kris

Davies, Simon

Davison, Glen

Day, Trevor

de Seigneux, Sophie

Debevec, Tadej

Degens, Hans

Dekerle, Jeanne

Del Re, Dominic

Dennery, Phyllis

Denton, Jerod

Desir, Gary

Desyatova, Anastasia

Deuis, Jennifer

Deymier, Alix

Dibb, Katharine

Dibner, Charna

Dickinson, Jared M.

Dinas, Petros

Dominelli, Paulo

Dorfman, Verónica

Doris, Peter

Dougherty, Kimberly

Drake, Amanda

Drew, Brian

Drust, Barry

Dube, John J.

Dudek, Edward

Dunyach‐Remy, Catherine

Duysens, Jacques

Dyck, David

Edwards, Aurélie

Egaña, Mikel

El All, Henrik

Elased, Khalid

Enoki, Taisuke

Epro, Gaspar

Essop, M. Faadiel

Evans, Roger

Faconti, Luca

Fadel, Paul

Faiss, Raphael

Falcone, John

Falgairolle, Melaine

Falz, Roberto

Faraci, Frank

Faulkner, Jessica

Figliuzzi, Marina

Filigheddu, Nicoletta

Fink, Julus

Fiorotto, Romina

Flanagan, Shawn

Florek, Ewa

Fonkoue, Ida

Foong, Jaime

Franchi, Martino

Frangogiannis, Nikolaos

Frump, Andrea

Fuentes, Nathalie

Fujimori, Ko

Fuloria, Mamta

Funai, Katsu

Gagnon, Daniel

Galea, Gabriel

Gallina, Alessio

Gamboa, Jorge

Gangula, Pandu

Ganz, Tomas

Gartman, Eric

Gatterer, Hannes

George, Sharon

Gerthoffer, William

Giacca, Mauro

Gibala, Martin

Gibson, Ann

Gironacci, Mariela

Gizzi, Alessio

Gonzalez, Liara

Goodman, Craig

Gorassini, Monica

Gorr, Matthew

Goswami, Nandu

Gottlieb, Lisa

Gravel, Hugo

Greaney, Jody

Greenwood, Iain

Griffin, Tomas

Grospretre, Sidney

Grubb, Søren

Guerrero, Francois

Hackett, Peter

Hackney, Anthony C.

Hajny, Stefan

Hamrefors, Viktor

Hansen, Clint

Harrington, Elizabeth

Harris, Robert

Harris, Ryan

Hashimoto, Takeshi

Hassanpour, Shahin

Hasson, Rebecca E.

Haus, Jacob

Heckman, Charles

Heinchelkan‐Martinez, Sergio

Hellsten, Ylva

Hennigar, Stephen

Heusser, Michelle

Hill, Warren

Hillebrandt, David

Hirai, Daniel

Hirata, Kosuke

Hjorth, Marit

Hoiland, Ryan

Holloway, Graham P.

Holm, Ninna Rose

Holwerda, Seth

Hook, Michelle

Horiuchi, Masahiro

Hou, Miao

Howarth, Frank

Hsia, Connie

Hu, Qinghua

Huang, Yu

Hughey, Rebecca

Hunter, Stacey

Hussain, Sabah

Hyldahl, Robert

Ichikawa, Hiroshi

Iemitsu, Motoyuki

Ikeda, Youko

Imig, John

Ionta, Silvio

Irvin, David

Isaksen, Jonas

Isanejad, Masoud

Iwanejko, Lesley

Jacobson, Jeffrey

Jamieson, Amanda

Jankowich, Matthew

Janssen, Paul

Jenkins, Nathaniel

Jepps, Thomas A.

Jernigan, Nikki

Jetton, Tom

Ji, Xiangming

Jobe, Alan

Johnson, Daniel

Kalantari, Naser

Kamijo, Keita

Kanawar, Yash

Kanno, Yoshihiko

Karlstaedt, Anja

Kasai, Takatoshi

Kashlan, Ossama

Kassab, Ghassan

Katakam, Prasad

Kaushik, Radhey

Kawada, Toru

Kearney, Mark

Kemp, Graham

Kennedy, David

Khan, Naim

Khandoker, Ahsan Habib

Kiselev, Anton

Kitta, Takeya

Klein, Janet

Knikou, Maria

Koike, Chieko

Kolwicz, Stephen C.

Kraft, Bryan

Kramer, Jessica

Krenn, Matthias

Krüger, Karsten

Kubo, K.

Kuperman, Yeal

Kurgan, Nigel

Kurtz, Ira

Kyaw Tun, Tommy

Lahm, Tim

Laird, Elizabeth

Lammers, Wim

Larsen, Steen

Laukkanen, Jari

Lavery, Gareth

Layden, Brian

Lederer, Eleanor

Leffert, Wesley

Legget, Kristina

Lelourd, Hugues

Levinthal, David

Li, Jennifer

Limberg, Jacqueline

Linkermann, Andreas

Liu, Ruisheng

Loenneke, Jeremy P.

Loewen, Matthew

Louis, Julien

Lovering, Andrew

Low, David

Lu, Qing

Lubberding, Anniek

Lund, Morten

Lustgarten, Michael

Machado, Aryane

Machida, Shuichi

Macintosh, Brian

Magness, Ronald

Majumdar, Adhip

Malin, Steven

Marcondes, Fernanda

Marshall, Janice

Martin, Brian

Massett, Michael

Masuda, Takahiro

Matsuyama, Shuichi

Mccafferty, Dominic

Mccarthy, Careron

Mccool, Dennis

Mcdaid, Liam

Mcgarr, Gregory

Mckeever, Kenneth

Mclean, Kelley

Mcloon, Linda

Mcmorris, Terry

Mcnamara, James

Mcpeek, Ashley

Mehrad, Borna

Mehta, Nehal N.

Mendelsohn Curanaj, Felicia

Meng, Lingzhong

Mentzer, Sj

Mermier, Christine

Michel, Martin

Millar, John

Millard‐Stafford, Mindy

Miller, Rachel

Millet, Grégoire

Mizuno, Masaki

Mohandas, Rajesh

Mohr, Maurice

Montain, Scott

Moo, Eng Kuan

Moon, Jordan

Moore, Daniel

Moore, Isabel

Morgan, Terry

Mori, Marcelo

Moro, Cedric

Morrison, Janna

Moss, Marcia

Muller, Matthew

Mullie, Patrick

Murfee, Walter

Murphy, Eric

Mutchler, Stephanie

Naeije, Robert

Nakamura, Fabio

Nandi, Shyam

Natali, Maria Raquel

Nelin, Leif

Nelson, Michael

Neubauer, Oliver

Newcomb, Dawn Catherine

Ng, Sean

Nightingale, Tom E.

Nissen, Sarah Dalgas

Nizar, Jonathan

Nolin, Thomas

Nomura, Seitato

Nord, Andreas

Nørregaard, Rikke

Nozik‐Grayck, Eva

Nunes, Everson

Nwosu, Benjamin

Nystoriak, Matthew

O'connor, Paul

O'reilly, Michael

Ogborn, Dan

Ogoh, Shigehiko

Okutsu, Mitsuharu

Opdenakker, Ghislain

Otto, Cynthia

Painelli, Vitor

Palmer, Lawrence

Palygin, Oleg

Pao, Alan

Papaioannou, V. E.

Parcell, Allen

Park, Jeanie

Patel, Neil

Pathare, Ganesh

Pearcey, Greg

Peng, Ying‐Jie

Penko, Amanda

Penton, David

Perkin, Oliver

Perry, Blake

Perry, Chris

Pesta, Dominik

Pethick, J.

Piknova, Barbora

Pinsky, Michael

Pires, Paulo

Ponnusamy, Murugavel

Pontzer, Herman

Poole, David

Porszasz, Janos

Porta, Alberto

Potter, Adam

Prado‐Garcia, Heriberto

Prakash, Y. S.

Price, S. Russ

Prisby, Rhonda

Przyklenk, Karen

Puricel, Serban‐George

Purves, J. Todd

Qiu, Xiaozhong

Quindry, John

Quinton, Paul

Rabchevsky, Alexander

Radak, Zsolt

Raine, Lauren

Ranadive, Sushant

Rao, Mrinalini

Rao, Rohit

Rasmussen, Peter

Ravn, Hanne Berg

Ray, Evan

Reckelhoff, Jane

Reynolds, Leryn

Rice, Charles

Ricoy, Ulises

Roberts, Michael

Robertson, Andrew

Robinson, Austin

Roche, Joseph

Rodgers, Kathleen

Rogers, Natasha

Rognant, Salomé

Romer, Shannon

Rondon‐Berrios, Helbert

Roper, Randall

Rosenberg, Alexander

Rounds, Sharon

Rowe, Glenn

Roxburgh, Brendon

Rubenstein, Ronald

Ryan, Michael

Ryan, Terence

Rynders, Corey

Sadeghi, Niloofar

Sadighara, Parisa

Saenz, Jose

Salinas, Michael

Saljic, Arnela

Salloum, Fadi

Samora, Milena

Samuelsen, Chad

Sandberg, Frida

Sands, Jeff

Sangha, Gurneet

Sapp, Ryan

Sasaki, Ryoki

Sassi, Yassine

Sattler, Stefan M.

Schebb, Nils Helge

Schertzer, Jonathan

Schmitt, Angelika

Scholkmann, Felix

Schubert, Rudolf

Schubert, Rudolf

Schultz, Jo El

Schur, Ellen

Secomb, Timothy

Semvelyan, Jasmine

Seo, Dae Yun

Seya, Yasuhiro

Sheldon, Iain

Shen, Bing

Shenkman, Boris

Shimoda, Larissa

Shiva, Sruti

Shiwarski, Daniel

Shoemaker, Leena

Siamwala, Jamila

Siddall, Andy

Siebenmann, Christoph

Sims‐Lucas, Sunder

Siracusa, Julien

Siu, Parco

Skytioti, Maria

Small, Deena

Smith, Caroline

Smith, Ian

Soleimani, Maryam

Sorensen, Charlotte

Sorrentino, Andrea

Spradley, Frank

Stampas, Argyrios

Stanhewicz, Anna

Stauss, Harald

Stavres, Jon

Steenbergen, Rineke

Stevens, Troy

Stierwalt, Harrison

Stokes, Keith A.

Straub, Adam

Subhan, Feisal

Subramanya, Arohan

Sullivan, Brynne A.

Sullivan, Maryrose

Sullivan, Millicent

Sun, Jun

Suzuki, Junichi

Sweeney, Gary Sweeney

Swenson, Eric

Swenson, Erik

Szczesna‐Cordary, Danuta

Taccola, Giuliano

Tamagnini, Francesco

Tan, Rachel

Tan, Roderick

Tan, Xiao‐Di

Tauscher, Markus

Taylor, Victoria

Thews, Oliver

Thoreson, Wallace

Thorin, Eric

Toledo, Frederico

Tomczak, Corey

Toth, Peter

Treebak, Jonas

Trigatti, Bernardo

Trittmann, Jennifer

Tsan, Linda

Tsuchimochi, Hirotsugu

Tucker, Torry

Ullah, Md Mahbub

Uwiera, Richard

Van Der Wijst, Jenny

Van Helden, Dirk

Veeraraghavan, Rengasayee

Veldhuizen, Ruud

Ventetuolo, Corey

Verges, Samuel

Vianna, Lauro

Vieira, Taian

Vijaya, Majumdar

Vincent, Philippe

Vincentelli, André

Viscor, Ginés

Vlahos, Ross

Voelkers, Mirko

Vogel, John

Vohwinkel, Christine

Volke, Vallo

Voynow, Judith

Wagner, Peter

Wall, Susan

Wallace, Darren

Wallis, Gareth Anthony

Wang, Jie

Wang, Xuejun

Wang, Xuexiang

Waters, Chrstopher

Webb, Clinton

Welch, Martha G.

Wernbom, Mathias

Whelan, Patrick

White, Danny

Whittle, John

Whysall, Kasia

Willems, Mark

Williams, Adam

Williams, Ian

Wilson, Thad

Witczak, Carol

Witman, Melissa

Wolf, Martin

Wood, Charles

Wright, Chris

Wright, David

Wu, Tongzhi

Xie, Chao

Yabe, Hiroki

Yang, Guang‐Yu

Yao, Hongwei

Yarar‐Fisher, Ceren

Yee, Jerry

Yokota, Takashi

Yoshihara, Toshinori

Yoshihisa, Akiomi

Yoshimura, Naoki

Yu, Xijie

Yuan, Yuhong

Zabbarova, Irina

Zeglinski, Matthew

Zera, Tymoteusz

Zhang, Dingmei

Zheng, Lei

Zhou, Lili

Zhou, Yang

Zimmer, Philipp

Zimmermann, Robert

